# Introgression and Divergence in a Young Species Group

**DOI:** 10.1111/mec.70448

**Published:** 2026-06-26

**Authors:** I. Satokangas, S. H. Martin, B. Seifert, T. Puukko, R. Schultz, H. Helanterä, J. Kulmuni

**Affiliations:** ^1^ Organismal & Evolutionary Biology Research Programme University of Helsinki Helsinki Finland; ^2^ Institute of Ecology and Evolution Ashworth Laboratories, University of Edinburgh Edinburgh UK; ^3^ Department of Entomology Senckenberg Museum für Naturkunde Görlitz Germany; ^4^ Ecology and Genetics Research Unit University of Oulu Oulu Finland; ^5^ Tvärminne Zoological Station University of Helsinki Hanko Finland; ^6^ Institute for Biodiversity and Ecosystem Dynamics, Department of Evolutionary and Population Biology University of Amsterdam Amsterdam the Netherlands

**Keywords:** divergence, *Formica* wood ants, hybridization, introgression, speciation

## Abstract

The process of speciation concerns often multiple diverging and interacting taxa, not only taxon pairs. Understanding the evolution of species' diversity and their persistence therefore requires insight into how gene flow and evolution of reproductive isolation shape groups of closely related species. Using resequencing data, we disentangle genomic patterns of divergence and introgression in five *Formica rufa* group wood ant species at an early stage of speciation. We revise earlier mitochondrial phylogenies using a nuclear genomic tree and demonstrate that introgression has occurred in line with observations of current day natural hybridisation. Genome‐wide correlations between divergence, differentiation, and diversity are in line with theoretical expectations for young species. However, despite previous evidence for polygenic species barriers, our data lack the genome‐wide correlation between differentiation and divergence expected under a polygenic barriers model. This is likely explained by the dominating effect of ancestral diversity at these early stages of speciation. Interestingly, we find no strong positive genome‐wide correlation between introgression and recombination, suggesting introgression is not predominantly deleterious. This contrasts with many previous empirical studies but aligns with new theoretical work on the interplay of divergence and introgression. Moreover, we observe low diversity in genomic regions with high introgression. This may signal that selection has favoured introgression in specific genomic regions. Such selection would be consistent with previous reports on wood ant hybrids, in which parental species ancestry is sorted likely due to positive selection, or because it allows reduction of genetic load. Benefiting from our focus on the whole *F. rufa* group and its introgression patterns, we show that interestingly, gene flow could cross multiple species boundaries even without direct interbreeding between each of the species. We discuss the long‐term benefits and costs of introgression in young species, including the effect of environmental fluctuations and multi‐species introgression.

## 1 Introduction

Recent studies have highlighted that speciation is a reticulate process that involves and is influenced by hybridisation and gene flow, the transfer of genetic material between diverging lineages. With repeated backcrossing, gene flow eventually leads to introgression, whereby genetic material from one species is transferred into and modifies the gene pool of another species. An understanding of the genomic signatures left by neutral, beneficial, or deleterious introgression can help to understand how it facilitates or slows down divergence and adaptation of incipient species (Peñalba et al. 2024; Abbott et al. 2013). A range of empirical examples show how gene flow and introgression may occur between multiple diverging lineages (Suvorov et al. 2022; Meier et al. 2023; Kozak et al. 2021), transferring genetic material even between species which do not directly interbreed with each other (Grant and Grant 2020). Despite this evidence, unanswered questions remain on how introgression influences speciation, especially in groups of young taxa.

Currently, it remains unclear how the fitness effects of introgression change during the process of divergence. Conventionally, introgression has been seen as largely deleterious, yet there is a growing body of evidence for introgression of adaptive alleles in various organisms (Oziolor et al. 2019; Norris et al. 2015; Racimo et al. 2015; Pardo‐Diaz et al. 2012). According to the conventional view, introgression is expected to be most prevalent in regions of high recombination, where adaptive or neutral alleles can be recombined out of a mostly deleterious genetic background and hence maintained in the population (Barton and Bengtsson 1986; Aeschbacher et al. 2017). Empirical studies of relatively strongly diverged species support these predictions (Sankararaman et al. 2014; Martin et al. 2019; Schumer et al. 2018; Juric et al. 2016).

Only recent theoretical work has addressed the interplay of divergence and introgression, especially how the relationship between recombination and introgression is predicted to change with divergence (Dagilis and Matute 2023). The fate of introgressed haplotypes is typically considered in terms of the combined fitness effects of linked alleles within genomic blocks (e.g., Sachdeva and Barton 2018). Extending this framework, Dagilis and Matute (2023) show that interactions among alleles within such blocks can play an important role. In contrast to the conventional view (Barton and Bengtsson 1986; Aeschbacher et al. 2017), they suggest that early in divergence introgression may be largely independent of recombination rate (Dagilis and Matute 2023). This arises because large introgressing haplotypes can carry coadapted sets of alleles with positive epistatic interactions. In young species, the benefits of such interactions may outweigh the costs of weak incompatibilities with the recipient genome. As a result, introgression can even be elevated in regions of low recombination (Dagilis and Matute 2023). Empirical patterns consistent with this prediction have been demonstrated, particularly in secondary contact, in *Drosophila melanogaster* (Duranton and Pool 2022; Pool 2015; Dagilis and Matute 2023) and European crows (Gwee et al. 2025). By contrast, if divergence occurs with continuous gene flow, species are expected to become and remain genomically distinct through adaptive divergence (see, e.g., Malinsky et al. (2015)), whereby introgression of diverged haplotype blocks would be maladaptive.

An alternative explanation for selection favouring introgression—in the absence of adaptive effects—is related to the impact of genetic load for introgression (Harris and Nielsen 2016). In these models, introgression masks (or purges) accumulated recessive deleterious variation in species with high genetic load. As a result, increased introgression can occur in the complete absence of even weakly beneficial introgressing alleles. This happens particularly in low‐recombination regions where linked selection is stronger, thereby generating a negative correlation between recombination and introgression (Kim et al. 2018). In this work, we use the term adaptive introgression both when introgression leads to beneficial exchange of genetic material that underlies differential adaptation, as well as when introgression is selected for because it masks deleterious recessive alleles, or facilitates the purging of genetic load. A signature of adaptive introgression is in both cases, at simplest, reduced nucleotide diversity (*π*) at regions of high introgression. This can signal that selection has favoured introgression as demonstrated, for instance, in *Arabidopsis* (Arnold et al. 2016). An exception is when the introgressed material carries deleterious recessive alleles at different sites in comparison to the recipient genome and can cause elevated heterozygosity (Harris and Nielsen 2016).

In this work, we measure genetic diversity as nucleotide diversity, *π*, the average number of nucleotide differences per site within a species. By genetic divergence we mean *d*_xy_, the average number of nucleotide differences per site between species (Nei and Li 1979). Differentiation, on the other hand, refers to *F*_ST_, which measures how similar or different allele frequencies are between two species (differentiation between species relative to the total genetic diversity across both species) (Weir and Cockerham 1984). These measures are expected to develop different correlations depending on the evolutionary scenario and the divergence between the species. In young species, sequence divergence (*d*_xy_) largely reflects the ancestral diversity (i.e., existing diversity (*π*) in the ancestral population before the incipient species became separated). The ancestral diversity varies heterogeneously along the genome due to background selection and local mutation rates (Charlesworth et al. 1993). Both diversity and divergence are computed from pairwise sequence differences. Therefore, in young species, regions with low within‐species diversity tend to also have low between‐species divergence, and those with high diversity have high divergence, creating a strong positive whole‐genome correlation between *π* and *d*_xy_ that serves as a hallmark of recently split species. Through time, the correlation weakens, as ancestral diversity becomes an increasingly small proportion of total between‐species divergence, and a negligible contributor to within‐species diversity. This will be exacerbated if patterns of within‐species diversity change as both lineages start experiencing increasingly different selection pressures. In the face of strong gene flow, even a negative correlation between diversity and divergence may emerge, as divergence accumulates in genomic regions where divergent selection limits the effective migration rate (Shang et al. 2023; Cruickshank and Hahn 2014). The relationship between genetic diversity and differentiation (*F*_ST_), on the other hand, may be stochastic in early divergence. In incipient species, initial differentiation can arise due to differences in sorting of the ancestral variation (i.e., a sampling effect). Over time, both positive and background selection are expected to lead to a negative genome‐wide correlation between differentiation and diversity that strengthens with increasing divergence (Burri 2017; Stankowski et al. 2019).

As divergence progresses, the amount of introgression decreases (Hamlin et al. 2020) while the prevalence of regions that resist it and can drive speciation in the face of gene flow, so‐called gene flow barriers, increases (Orr and Turelli 2001; Orr 1995). However, how their genomic distribution develops, or whether they are formed of a few regions or scattered along the whole genome has remained under investigation (Laetsch et al. 2023; Wu 2001). ‘Outlier scans’ have been commonly used to detect highly differentiated regions that supposedly arise because they resist gene flow. Specifically, it is suggested that gene flow barriers should manifest as regions where both differentiation and absolute divergence (*d*_xy_) are elevated (Cruickshank and Hahn 2014).

We study introgression and divergence using *Formica rufa* group wood ants that have diverged recently within the last ~500.000 years (Portinha et al. 2022; Goropashnaya et al. 2004). They live in boreal forests throughout Eurasia with large sympatric areas (Stockan and Robinson 2016). The *F. rufa* group has at least two distinguishable features in which the species differ from each other and that likely contribute to their speciation. First, the sister species harbour different social strategies (polygyny and monogyny) that are coupled with their dispersal patterns and habitat requirements (Seifert 2018; Stockan and Robinson 2016). Second, the non‐sister species have different climatic adaptations that are reflected in their geographical distributions (Stockan and Robinson 2016). At least two non‐sister species have, according to demographic modelling, diverged with continuous gene flow until recent times (Portinha et al. 2022). However, as the *F. rufa* group species have been suggested to diverge for periods of time in separated forest regions, the Pleistocene glacial refugia (Goropashnaya et al. 2004), their history likely encompasses both periods of allopatry and gene flow. Currently multiple species in the *F. rufa* group hybridise extensively (both sister‐ and non‐sister species), which has led to formation of further generation and backcrossed hybrid populations (Satokangas et al. 2023; Seifert 2021). Hybridisation of the two non‐sister species, *F. aquilonia* and *F. polyctena* , has been coupled with various consequences. These include hybrid mortality (Kulmuni and Pamilo 2014), repeatable sorting of ancestry in replicate hybrid populations presumably due to both genetic load and favouring of adaptive alleles (Nouhaud, Martin, et al. 2022), and potential to adapt to climate change due to differential climatic adaptations (Satokangas et al. 2023; Martin‐Roy et al. 2021). However, the overall fitness effects and long‐term impacts of introgression are not known, or how speciation and polygenic gene flow barriers detected in previous work (Kulmuni and Pamilo 2014; Kulmuni et al. 2020; Heidbreder et al. 2026) manifest as whole‐genome divergence in this group.

Using a comparative population genomic framework, we utilise new and pre‐existing resequencing data from five wood ant species to understand how different processes have contributed to their speciation during the recent divergence history. We demonstrate that genetic divergence resembles ancestral diversity, signalling recent speciation. We revise earlier mitochondrial phylogenies with a nuclear species tree. Despite a high proportion of unsorted variation, the tree clusters individuals by species regardless of geographic origin. We find that genome‐wide correlations between population genetic parameters are in line with theoretical expectations for young species; there is a strong significant positive correlation between divergence and within‐species diversity. We detect introgression in both sister and non‐sister species pairs, yet there is no signal of barriers to gene flow that would be detectable as a correlation between differentiation and divergence at the whole‐genome scale. In previous work, recent‐day hybrids have been shown to suffer from mortality, but they are also suggested to benefit from decreased genetic load and increased potential to adapt to changing climate. Our results on the introgression landscape suggest that the overall inferred introgression is largely neutral or even beneficial, following the expectations for young species from new theoretical work. Neutrality is inferred from at most a very weak positive whole‐genome correlation between recombination and introgression. Low diversity in the regions of highest introgression indicates a proportion of introgression may be adaptive, in line with previous work on wood ant hybrids suggesting heterospecific ancestry may mask recessive genetic load, or bring other adaptive value.

## 2 Materials and Methods

### 2.1 Sampling

We studied genomic signatures of diversity, differentiation, divergence, and introgression among five *Formica rufa* group wood ant species. Our aim was to investigate if we found signatures of either adaptive or deleterious introgression among the species group. We also studied the relationship between diversity, divergence, and differentiation to produce a thorough understanding of speciation in this group, and constructed an improved whole‐genome species tree.

In previous work, we have extensively studied the hybrid wood ant populations (Kulmuni et al. 2010, 2020; Kulmuni and Pamilo 2014; Nouhaud, Martin, et al. 2022; Heidbreder et al. 2026; Satokangas et al. 2023; Krapf et al. 2025). We have shown that the hybrids are abundant and persist in nature over decades, often tending towards balanced admixture proportions rather than spanning the full range. In the current project, we therefore focused on non‐admixed populations to study whether the extensive hybridisation also results in introgression between the species.

For these purposes, we sampled individual wood ant workers from five sympatrically living *Formica rufa* group species: *F. aquilonia* , *F. lugubris* , *F. polyctena* , *F. rufa* , and *F. pratensis* (Satokangas et al. 2023; Stockan and Robinson 2016). The majority of the samples originate from Finland, supplemented with samples from Central Europe and Scotland to offer a broader perspective.

Mostly one individual per colony was used, each corresponding to a unique population (with minimum distance between colonies six kilometres, except for two *F. aquilonia* colonies 2.6 km). This sampling aimed to facilitate covering as many genomically distinct populations as possible within the known distribution of each species in Finland (Stockan and Robinson 2016). As some of our study species are ‘supercolonial’ (Helanterä 2022), that is, several nests within populations may comprise large genetically homogeneous societies, any additional individuals from the same population would not be genetically independent replicates, but relatives. Furthermore, differences in dispersal and relatedness structures between the species would mean that the problems arising from non‐independence would be different in magnitude for different species if multiple individuals per population were sampled (Sundström et al. 2005).

We utilised whole‐genome sequencing data, most of our data consisting of individuals sequenced in previous work (Satokangas et al. 2023; Portinha et al. 2022). In addition to *F. rufa* group samples, *Formica exsecta* , a distantly related species belonging to the *Formica exsecta* group (Borowiec et al. 2021), was used as an outgroup in some analyses (Nouhaud, Martin, et al. 2022); originally published in Dhaygude et al. (2019). New *F. rufa* group ant samples with no published genomic data were obtained from Germany and Switzerland, primarily to clarify the relationship between *F. polyctena* and *F. rufa* . These samples were stored in ethanol prior to DNA extraction and classified morphologically (later confirmed with genomic identification). This morphological classification was based on investigation of dry, mounted worker specimens by high‐resolution stereomicroscopy considering 16 characters. These characters included one indicator of absolute size, four shape variables, seta counts in six body parts and seta length measurements in five body parts. In order to improve the performance of a principal component analysis removal of allometric variance was performed for all shape and seta characters for the assumption of each individual having a cephalic size of 1750 μm (for details see Seifert (2021)). See Table S1 for more information on the sampling, including sampling locations and years. In this study, a total of 93 specimens from the *F. rufa* group were analysed (45 × *F. aquilonia* , 8 × *F. lugubris* , 6 × *F. polyctena* , 7 × *F. pratensis* , 9 × *F. rufa* , 18 × admixed). The majority of the analyses were performed with five individuals per species, while the remaining individuals served as context for our findings. This research complies with all applicable laws on sampling from natural populations.

### 2.2 Whole‐Genome Sequencing

For the individuals used in previous work, the details of whole‐genome sequencing and first data processing steps can be found in Satokangas et al. (2023). For the new samples, we extracted DNA from the whole worker ant bodies. Tissue samples (individual workers) were stored in ethanol. Before genomic DNA extraction we removed the ethanol by gently pressing with a paper towel and then we ground the samples in micro centrifuge tubes using liquid nitrogen and plastic pestles. We then extracted DNA with Qiagen's DNeasy Blood & Tissue Kit (Cat. No./ID: 69504). We extended the lysis step overnight and did not do the RNase treatment. We quantified DNA concentrations with a ThermoFisher Scientific Qubit DS DNA Kit (Q32851).

We randomised the sample order and then used New England Biolabs NEBNext Ultra II FS DNA Library Prep Kit for Illumina (E7805L) to prepare the DNA libraries, and indexed the samples using NEBNext Multiplex Oligos for Illumina Dual Index Primers Set 1 (E7600S). We followed the manufacturer's protocol for use with inputs ≥ 100 ng DNA and we used 150 ng of DNA for each library preparation. We aimed for the fragment size range at 200–450 bp; to achieve this we had to reduce the recommended incubation of 15 min at 37°C to 11 min. We used Beckman Coulter Life Sciences AMPure XP SPRI Reagent (A63881) for the steps requiring magnetic beads and the magnet was ThermoFisher Scientific Invitrogen magnetic stand‐96 (AM10027). Our aim for the final size of DNA libraries was approximately 320–470 bp. We verified that and the lack of impurities with Agilent 5200 Fragment Analyzer and ProSize data analysis software. We measured DNA library concentrations with the ThermoFisher Scientific Qubit DS DNA Kit (Q32851).

Once the DNA libraries had passed our quality control, we prepared two pre‐pools from individual library samples, one containing three samples with average library size ranging from 320 to 396 bp and the other containing nine samples with average library size ranging from 486 to 579 bp. Equimolar amounts of individual DNA samples were used, based on the library average size data. Finally, we sent these pre‐pools on dry ice to Novogene Corporation Inc. (United Kingdom), and they performed the Illumina NovaSeq sequencing runs with 150‐base‐pair paired‐end reads.

We trimmed the raw Illumina reads and adapter sequences with Trimmomatic v0.39 (Bolger et al. 2014) and then mapped them to a F. aquilonia × F. polyctena hybrid reference genome (Nouhaud, Beresford, and Kulmuni 2022) with BWA‐MEM algorithm in BWA v0.7.17 (Li 2013). We removed duplicates with Picard tools v2.21.4 (http://broadinstitute.github.io/picard) and clipped overlaps with BamUtil v1.0.15 (https://github.com/statgen/bamUtil). Use of the hybrid reference genome is unlikely to have biased our results, as mapping rates were high across all species (see Table S2; mean rates: *F. aquilonia* 98.10%, *F. lugubris* 99.44%, *F. polyctena* 98.58%, *F. pratensis* 99.54%, and *F. rufa* 99.27%); consistent with earlier work (Satokangas et al. 2023; Nouhaud, Beresford, and Kulmuni 2022).

These bioinformatic steps had been performed separately also for the previously published data using the same pipeline (Portinha et al. 2022; Satokangas et al. 2023), except for overlap clipping that had not been performed for samples from Portinha et al. due to low fraction of read overlap (4% in Portinha et al. vs. 13%–15% in the other samples). For the previously published data used in this study, average read depth was 16.0× (Satokangas et al. 2023). Our average read depth for the newly sequenced individuals was 10.5× (for individuals that passed all filters and were analysed in this study; see Table S3), computed with *mosdepth* v0.3.3 (Pedersen and Quinlan 2018).

We performed the following steps for all samples simultaneously. Variants were called for the nuclear genome excluding short genomic regions with no known location (“Scaffold 00” in the genome assembly), with *FreeBayes* v1.3.6, using ‐k option to disable population priors (Garrison and Marth 2012). We normalised the vcf file with *VT* v0.57721, (Tan et al. 2015). We then filtered out sites at two base pair distance from indels, as well as sites that were supported by only forward or reverse reads, with *BCFtools (*Danecek et al. 2021). We decomposed multinucleotide variants using vcfallelicprimitives from *vcflib* v1.0.0_rc3 (Garrison et al. 2022). We filtered the resulting vcf file with *BCFtools* to keep only biallelic single nucleotide polymorphisms (SNPs) with quality equal or higher than 30. We then identified and set missing all sites that had over two times the mean sequencing depth of each individual in question. The excess depth may be an indication of collapsed gene duplications in the reference genome. We further removed sites with heterozygote excess when all samples were pooled, as these sites may represent genotyping errors for example due to misaligned reads (*p* < 0.01, *VCFtools* v0.1.16 (Danecek et al. 2011)). We set individual genotypes that had < 8 coverage as missing.

We excluded seven individuals with more than 50% missing data and three additional *F. rufa* group individuals that were sequenced for collaborative purposes and excluded then sites that had more than 10% missing data over all remaining individuals. We also removed Scaffold 03, the so‐called social chromosome with large inversions (Brelsford et al. 2020) as it would bias further computations (yet analysed in other recent work, see Sigeman et al. (2024)), and excluded singletons (minor allele count < 2) with *VCFtools*. Extra filtering steps required for some of the analyses that we performed are mentioned with each analysis' description.

As a result, our dataset (the “main vcf” from here onwards) for this study comprised 93 *F. rufa* group individuals with 1.890.044 biallelic SNPs.

### 2.3 Genetic Structure

To confirm the genetic clustering and species identity of the new morphologically identified samples, we recreated the phylogenetic network from Satokangas et al. (2023). Especially, our new *F. rufa* samples helped in confirming whether the closely related and interbreeding sister species *F. rufa* and *F. polyctena* cluster by species regardless of their geographic origin.

Using the main vcf (93 individuals) thinned such that there was at least 20 kb between each variant, resulting in altogether 9.816 biallelic SNPs, we computed pairwise genetic distances of all pairs of samples with distMat.py (github.com/simonhmartin/genomics_general), and generated a phylogenetic network from the resulting distance matrix using *SplitsTree* v4.17.1 (Huson and Bryant 2006) and the NeighbourNet approach (Bryant and Moulton 2004) with default parameters.

### 2.4 Outgroup

To construct and root a species tree and infer introgression in the *F. rufa* group (see below), we used *F. exsecta* as an outgroup. We utilised bam files mapped with the same reference genome as in this study (mapping quality ≥ 20, sequencing depth 14.5×), generated by Nouhaud, Beresford, and Kulmuni (2022). The sequencing data originates from Dhaygude et al. (2019) who had whole‐genome sequenced a pool of 50 haploid male ants with 100‐base‐pair paired‐end reads generated on Illumina (ENA accession number SAMN07344806).

We extracted the *F. exsecta* genotypes by calling variants at the biallelic SNP loci with *BCFtools* mpileup (to generate the genotype likelihoods, disabling the computation of base alignment quality and filtering out bases with quality less than 20) and *BCFtools* call (to perform the actual calling), setting ploidy to 1, and removing duplicate sites retaining one allele per locus. This pipeline was adapted from Nouhaud, Beresford, and Kulmuni (2022). See each analysis for when the outgroup SNPs were called and merged (with *BCFtools* merge) with the main vcf. The outgroup was merged in all cases after minor allele filtering, as the outgroup is represented only as one haploid genotype per locus.

### 2.5 Phylogenetics

To investigate the nuclear species tree of the *F. rufa* group, we constructed a whole‐genome neighbour‐joining (NJ) tree, supplemented by windowed gene tree inference. The NJ tree is suitable for very large datasets, and for both within‐ and between‐species trees as it does not force the data with any evolutionary assumptions. For gene trees, we examined the most frequent topology inferred from trees built from small genomic windows (individual genes). Only coding sequences were used, as these are expected to be under purifying selection. This helps mitigate the effects of incomplete lineage sorting (ILS) (Scally et al. 2012) and introgression (Martin et al. 2019), the impact of which is important to account for (Burbrink et al. 2025) when assessing the species tree.

#### 2.5.1 NJ‐Tree

For the NJ‐tree, we thinned the main vcf file such that we had a minimum 1 kb distance between each variant with *VCFtools*. We did thinning for both computational reasons but also because in this way regions of the genome which have a distinct history, like those under strong selection, impact the analysis less. We then called the outgroup *F. exsecta* variants and merged the outgroup vcf to the thinned main vcf. We selected individuals that clustered genomically together by species (Figure S1, and Satokangas et al. (2023): Figure 1) as including clearly admixed individuals would be unhelpful in constructing the species tree. We further excluded two individuals with more than 30% missing data to maximise the number of SNPs available for the tree, and as the last step excluded all SNPs with more than 1.0% of missing data over all individuals, retaining altogether 73 individuals (minimum six individuals per species) and the outgroup (See Table S1), and 165.332 SNPs.

**FIGURE 1:**
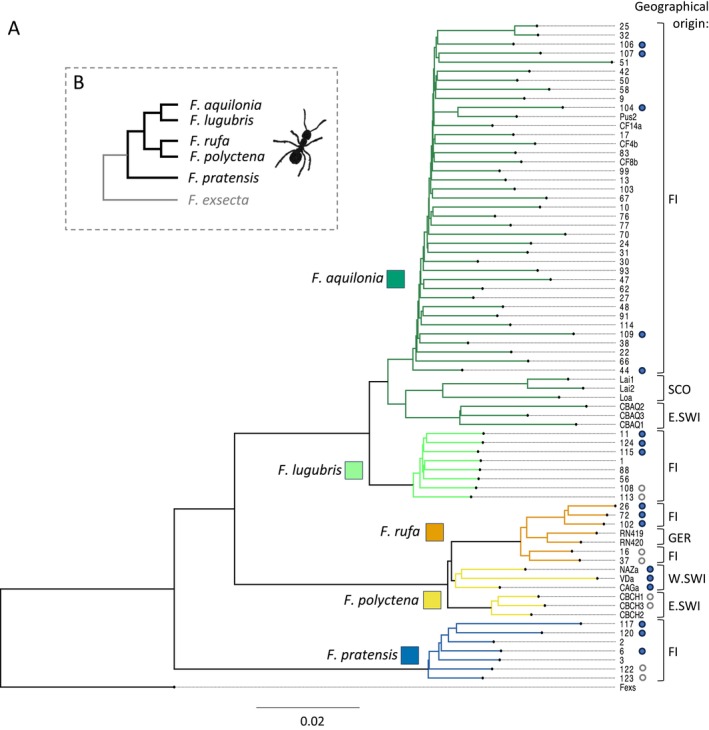
Species tree. (A) Neighbour‐joining tree based on whole‐genome data (73 individuals and the outgroup, 534.202 SNPs) shows that despite high amounts of unsorted genetic variation, all species but *F. polyctena* form monophyletic groups. The tree is rooted with *F. exsecta* (“Fexs”) and bootstrap support is 100 for all nodes (as percentages of 100 replications). Geographic origin of each individual is shown at the end of the branches. The branch length scale bar indicates the fraction of changed sites. Individuals with blue and white circles were used for all windowed analyses and *f*‐branch. The colour difference of the circles shows how the individuals were split in two groups for the second *f*‐branch run (Figure S2B). For this second run, part of the *F. aquilonia* individuals were replaced by Swiss and Scottish samples (Table S1); not shown here. (B) Inset schematic species tree showing the inferred species relationships (branch lengths are not to scale).

We then converted the vcf into phylip format with vcf2phylip script (v2.8, (Ortiz 2019)) with default parameters and *F. exsecta* defined as the outgroup. We constructed a distance matrix from the phylip file using the *phangorn* package (Schliep 2011) in R, with JC69 (Jukes and Cantor 1969) substitution model that assumes equal base frequencies and equal mutation rates. We then performed 100 bootstraps with *phangorn*, and visualised the tree using *Figtree* v.1.4.4 (http://tree.bio.ed.ac.uk/software/figtree/) rooting it with *F. exsecta* .

#### 2.5.2 Gene Tree Inference

For the gene tree inference, we used the main vcf with merged outgroup (no thinning). We used the same 73 non‐admixed individuals that were used for the NJ tree.

We first phased the SNP data with WhatsHap v2.0 (Martin et al. 2016) and then ShapeIt4 v4.2.2 (Delaneau et al. 2019) (parameters ‐‐mcmc‐iterations 10b, 1p, 1b, 1p, 1b, 1p, 1b, 1p, 1b, 1p, 1b, 1p, 10m ‐‐pbwt‐depth 8 ‐‐use‐PS 0.0001). For the duration of phasing, the outgroup *F. exsecta* was subsetted out from the dataset as it is haploid.

We then filtered the vcf to contain only coding sequence (CDS) regions, using our hybrid reference genome annotation file. We converted the cds‐only vcf to a geno file with parseVCF.py script (https://github.com/simonhmartin/genomics_general).

We extracted all gene regions from the reference genome annotation file, making a region file with one region per gene. We then built gene trees using phyml_sliding_windows.py script (https://github.com/simonhmartin/genomics_general), using the five *F. rufa* group species as ingroup taxa, and *F. exsecta* as an outgroup. We used the HKY85 substitution model (Hasegawa et al. 1985). No model parameters were estimated. We replicated the tree building twice, allowing only regions (windows) that had a minimum of (i) 20 or (ii) 50 variant sites. This resulted in 1475 and 200 windows, respectively.

We then examined the most frequent topology by using *TWISST*, ‘Topology Weighting by Iterative Sampling of Sub ‐Trees’ (Martin and Van Belleghem 2017). *TWISST* constructs subtrees by iteratively sampling one sample from each group at a time. It then quantifies the proportion of each possible tree topology (in our case possible topologies among the five species) in the given window. Finally, we summarised the genome‐wide proportions of all possible tree topologies to infer the most frequent topologies.

### 2.6 Tests of Divergence and Diversity

To investigate the genome‐wide patterns of speciation, we computed differentiation *F*_ST_, divergence *d*_xy_, and nucleotide diversity *π* in 100 kb genomic windows.

#### 2.6.1 All‐Sites vcf

For this purpose, we first generated an all‐sites vcf file for each scaffold separately with *BCFtools* v1.16 mpileup and *BCFtools* call commands with multiallelic call mode. To calculate a maximum allowed per‐site coverage, we utilised the individual mean depths that were computed in the main vcf pipeline for the same purpose. For all‐sites vcf, we set the maximum depth per site (over all individuals) as two times the mean depth of per‐individual averages. The maximum depth filtering was done to exclude collapsed paralogous genomic regions. We set a minimum depth per site over all individuals as two times the number of individuals (i.e., on average, two reads per individual). Sites not meeting these thresholds were set as missing. We then removed the same individuals that we removed from the main vcf, that had shown over 50% missing data, and excluded all sites that had more than 50% missing data. Next, from each scaffold‐specific vcf file, we extracted one vcf file with only invariant sites, and another one with only variant sites, with *VCFtools* v0.1.17. In each variant site vcf we kept only sites with minimum quality of 30 and excluded sites that showed excess heterozygosity (vcftools ‐‐hwe 0.001). Singletons were filtered out, as rare uninformative markers potentially bias (lower) *F*_ST_ values (Roesti et al. 2012). Finally, we combined all scaffolds and the invariant and variant site vcf files with *BCFtools* concat to get one all‐sites vcf file and removed duplicate sites. This resulted in an all‐sites vcf file with 194.332.157 sites and 93 individuals.

#### 2.6.2 *F*_ST_, *d*_xy_, and *π* Computation in Genomic Windows

We computed the *F*_ST_, *d*_xy_, and *π* statistics for 100 kb non‐overlapping windows, using the *PIXY* package (Korunes and Samuk 2021). *PIXY* was used as it accounts for missing data as this would otherwise lead to biassed estimates. Window size of 100 kb was chosen as it is large enough for liable *F*_ST_ estimates with low sample size (Nadeau et al. 2012). Each window contained loci from only one scaffold (no windows crossing scaffold boundaries). To reduce stochasticity, we excluded windows where data for more than half of the sites (50 kb) was missing, as well as windows with less than 100 SNPs. For species pairs' comparisons, mean *π* of both species in question was calculated for each genomic window. *F*_ST_ values below zero were converted to zero following a common practice. For *F*_ST_, *d*_xy_, and *π* computation we used five individuals per species with least missing data, each from a different population within Finland (as in *f*‐branch statistic below). For *F. polyctena* our sampling did not allow this, and we chose five that maximised the number of individuals coming from different populations in Switzerland (see Table S1).

### 2.7 Tests of Introgression

#### 2.7.1 Genome‐Wide Introgression

To test for signatures of introgression between the *F. rufa* group species, we computed the *f*‐branch (*f*_*b*_) statistic with *Dsuite* package (Malinsky et al. 2021). *f*_*b*_ is based on estimating the proportion of genome‐wide introgression (*f*)—the relative amount of shared variation between two non‐sister taxa P2 and P3, compared to P1 and P3, given altogether four taxa (((P1, P2), P3), Outgroup). *f*_*b*_ helps with the interpretation of correlated introgression proportions between related taxa by assigning gene flow not only to tips of a phylogeny but also to the internal branches.

We added the outgroup vcf to our main vcf with *BCFtools* merge. We used the NJ‐tree, gene tree, and genetic structure results from this study to provide a tree hypothesis and to group the individuals for the analysis. First, we selected five individuals per species with the least missing data and *F. exsecta* specified as the outgroup (Table S1) to compute *f*_*b*_ between the species (Figure S2A). This simplest tree, however, did not allow us to evaluate introgression between sister species *F. rufa* and *F. polyctena* , or *F. aquilonia* and *F. lugubris* . Hence, we computed the *f*_*b*_ statistic also for a more detailed tree (Figure S2B), where all species were separated into two groups based on the deepest split in the species tree (Figure 1).

The motivation for this is that, when introgression is recent or variable in space, individuals of the same species may differ in their proportion of introgression (Hamlin et al. 2020). Such differential introgression can drive phylogenetic clustering, so our decision to use the deepest split within each species to define two groups maximises our potential to detect distinct introgression proportions between the groups. Detection of such differences in introgression between a species and populations of its sister species would in itself be evidence of introgression between these two sister species.

Therefore, the second analysis was run with two to three individuals with the least missing data per group, determined by the availability of individuals per species. If the deepest split separated only one individual from the rest, a second individual was added using the same procedure, that is, after the second‐deepest split. For *F. aquilonia* , two individuals from Switzerland and one from Scotland were added for this detailed analysis. For *F. aquilonia* and *F. polyctena* , the within‐species split reflected differences in geographical origin.

#### 2.7.2 Introgression in Genomic Windows

We further investigated the genomic landscape of introgression, especially asking whether introgression is more prevalent in the regions of low diversity and recombination, consistent with signatures of adaptive introgression. For this, we computed the *f*_*d*_ statistic (Martin et al. 2015) in 100 kb non‐overlapping sliding windows with ABBABABAwindows.py script from https://github.com/simonhmartin/genomics_general. The *f*_*d*_ is derived from the standard estimator of the proportion of introgression (*f*), applied in *f*‐branch. For instance, *f*_*d*_ of 0.1 for a given window indicates that 10% of haplotypes in the recipient population in this genomic window are inferred to be introgressed.

While the standard *f* estimator is sensitive to stochastic errors when the number of studied variants is small (as in windowed analysis), *f*_*d*_ accounts for such stochastic noise by assuming the most conservative direction of introgression at each site (Martin et al. 2015). In other words, to compute the standard *f* statistic, the observed excess of shared derived alleles is divided by the expected excess under the maximum possible amount of introgression (complete ‘swamping’). The maximum possible introgression is conventionally estimated by treating P3 (the source population for observed introgression) as both the source and recipient population. When, at a specific site, the assumed recipient population (P2) has a higher frequency of the derived allele than the assumed source population (P3), this may lead to overestimation of windowed *f*‐values (*f* may exceed one at a specific site). To correct for this, when calculating the maximum possible introgression, *f*_*d*_ chooses for each site the source population based on which population (P2 or P3) has a higher frequency of the derived allele. This eliminates *f* estimates that exceed 1.

We estimated *f*_*d*_ between species pairs for which we identified genome‐wide introgression with the *f*‐branch statistic, that is from *F. polyctena* into *F. aquilonia* , from *F. aquilonia* into *F. polyctena*, and from *F. polyctena* into *F. rufa* (Table 1). Furthermore, we estimated *f*_*d*_ in three species pairs with no significant detected introgression at the genome‐wide scale using *f*‐branch to confirm that signal of high introgression is not an artefact caused by other genomic properties (Table 1). For studying introgression between *F. polyctena* and *F. rufa*, two or three individuals per group were used (following the same procedure as for *f*‐branch, described in 2.7.1), while for all other comparisons the number of individuals was five. It needs to be kept in mind that the test is always relative, limited to revealing variation shared by P3 and P2, but not P1. Hence, for instance, when inspecting gene flow between *F. rufa* and *F. polyctena* , we are able to detect only introgression that is shared by some *F. rufa* populations (P2) but not the others (P1).

**TABLE 1:** Species combinations used for studying introgression with *fd* statistics in 100 kb windows.

Studied introgression (number of individuals per group)	P1	P2	P3	Outgroup
pol → rufa (2 or 3)^a^	*F. rufa*	*F. rufa*	*F. polyctena*	*F. exsecta*
pol → aqu (5)^a^	*F. lugubris*	*F. aquilonia*	*F. polyctena*	*F. exsecta*
aqu → pol (5)^a^	*F. rufa*	*F. polyctena*	*F. aquilonia*	*F. exsecta*
prat → pol (5)	*F. lugubris*	*F. polyctena*	*F. pratensis*	*F. exsecta*
prat → aqu (5)	*F. lugubris*	*F. aquilonia*	*F. pratensis*	*F. exsecta*
lug → rufa (5)	*F. polyctena*	*F. rufa*	*F. lugubris*	*F. exsecta*

*Note:* pol = *F. polyctena* , aqu = *F. aquilonia* , rufa = *F. rufa* , lug = *F. lugubris* , prat = *F. pratensis*.

^a^ Significant introgression detected with the *f*‐branch statistic.

To quantify *f*_*d*_, windows with nominator values < 0 were converted to zero. The nominator quantifies the excess of derived alleles between P3 and P2, and for this statistic, negative values (that would indicate the excess between P3 and P1) are meaningless. Stochasticity of *f*_*d*_ values was minimised by using a 100 kb window size and retaining only windows with a minimum of 100 biallelic SNPs.

### 2.8 Population Recombination Rate

To investigate the relationship between introgression and recombination (see also Section 2.7.2), we used the population recombination rate (rho, *ρ*) estimates computed in previous work (Nouhaud, Martin, et al. 2022). The *ρ* estimates had been computed with *iSMC* software (Barroso et al. 2019) using six *F. polyctena* and six *F. aquilonia* individuals from Switzerland and Scotland (these individuals are also included in this study; see Table S1), for 20 kb non‐overlapping windows. These windows started from varying positions in different scaffolds, which is why we averaged the values over 100 kb non‐overlapping windows and 20 kb recombination windows spanning two 100 kb windows were counted in both.

When interpreting the correlations between introgression and recombination or diversity, it needs to be kept in mind that the *iSMC ρ* values and diversity (*π*) are correlated with each other as both are affected by the local effective population size N_e_. This is because *ρ* is a composite parameter that is scaled by N_e_ (population recombination rate *ρ* = 4N_e_*r, where *r* = recombination rate, and N_e_ is multiplied by four because it accounts for diploidy and recombination events in two lineages), and *π* is an estimator of the population mutation rate *θ* that is also scaled by N_e_ (*θ* = 4N_e_*μ, where *μ* = per‐generation mutation rate; Watterson 1975; Barroso et al. 2019). These estimators are affected only by those recombination events or mutations that have occurred more recently than the most recent common ancestor of the individuals in question. For this reason, low *ρ* and low *π* both occur in regions of low N_e_ regardless of the local crossover rate, which is why observations of low *ρ* and *π* may not be independent.

### 2.9 Genome‐Wide Correlations Between Population Genetic Parameters

After we had summarised the genomic variation in diversity (*π*), divergence (*d*_xy_), differentiation (*F*_ST_), population recombination rate (*ρ*), and introgression (*f*_*d*_) in 100 kb non‐overlapping sliding windows, we used Spearman's rank correlation to compute genome‐wide correlations between these statistics to investigate the genomic landscape. The correlations involving introgression were also tested with a window size of 50 kb to examine their robustness. Spearman's correlation suits our data as it does not assume a linear relationship between variables. We are aware that the 100 kb genomic windows are not independent data points because of genetic linkage of loci between the windows, and that this pseudoreplication may affect the correlation test *p*‐values. However, the correlation tests provide a valuable way to summarise genomic variation and compare empirical data with theoretical expectations.

To confirm that our correlation results were not affected by higher diversity caused by genomic regions that have been collapsed in the genome assembly and have remained in the data despite vcf filtering, we performed an additional filter step, where we excluded high‐coverage outlier windows from our correlation analyses. For this, we computed per‐species mean coverage in 100 kb windows from bam files using *mosdepth* v0.3.3 (Pedersen and Quinlan 2018), using the individuals for which the correlation analyses were run. We then removed all windows in which the coverage for any species exceeded two times the mean coverage of that species, thus excluding potentially biassed genomic regions.

We computed genome‐wide correlations between (a) population recombination rate and introgression (*ρ* and *f*_*d*_), (b) diversity and introgression (*π* and *f*_*d*_), (c) diversity and divergence (*π* and *d*_xy_), (d) diversity and differentiation (*π* and *F*_ST_), and (e) divergence and differentiation (*d*_xy_ and *F*_ST_), with the following expectations:

- *Positive correlation between recombination and introgression* is suggested to be indicative of dominating deleterious introgression, with a large number of barrier loci dispersed throughout the genome. Recombination is needed to separate beneficial or neutral introgressing material from the prevalent deleterious material leading to relative excess of introgression in high recombining regions (Veller et al. 2023). In low recombining regions the introgressed material with mostly deleterious haplotypes is selected against. In addition, many alleles with small deleterious effects can be jointly selected against more efficiently than in high recombining regions, as their selective coefficients are combined.
*An absence of correlation or negative correlation* is suggested to arise from neutral or beneficial fitness effects of large introgressing haplotypes, as the haplotypes may get selected for as a whole without the need of partitioning by recombination, or selection on the introgressed neutral haplotypes is not affected by recombination. This may reflect either that polygenic barriers (consisting of many loci) have not yet evolved, or that the direct fitness benefits of the introgressing alleles and coadaptation within the introgressing block outweigh or are equal to the negative epistatic interactions of introgression with the recipient genome.

b *Negative correlation between diversity and introgression* is consistent with adaptive introgression. This suggests that selection has favoured introgression and hence reduced diversity either because introgression transfers adaptive traits or because it may reduce genetic load in the recipient species. However, if both source and recipient species have low diversity in a region of high introgression, then the low diversity (and the correlation) may be caused by strong background selection that has persisted since before speciation, rather than by an adaptive introgression event.
c *Strong positive correlation between diversity and divergence* is expected in young species as sequence divergence between young species predominantly reflects ancestral diversity (diversity in the ancestral population that predated the speciation event) or the lack thereof. This correlation weakens over time as sequence divergence accumulates through mutation, drift, and selection. In the face of strong gene flow, divergence may only accumulate in genomic regions where divergent selection acts to limit the effective migration rate, which might eventually lead to a negative correlation between diversity and divergence.
d *The correlation between diversity and differentiation* may be stochastic in early divergence due to sampling effects and differences in sorting of ancestral variation, reasoned in a verbal model (Burri 2017). As differentiation increases, the effects of positive selection and later of linked selection (hitchhiking as well as background selection) start to be detectable. A negative whole‐genome correlation is expected to arise and strengthen through divergence: selection acts independently in both lineages, and where it has the strongest effect, it may lead to differential fixation (low diversity) and hence elevated differentiation.
e *Positive correlation between divergence and differentiation* is expected in divergence with gene flow if a large number of gene flow barriers are present throughout the genome.

### 2.10 Local Genomic Relationship of Introgression, and Diversity or Recombination

In addition to the correlation tests described above, we also performed post hoc tests for enrichment of introgression outliers in regions of low diversity or low recombination. A high proportion of introgression associated with low diversity and low recombination is a potential indication that this introgression is favoured by selection (see the expectations in Section 2.9).

For this, we first divided the 100 kb genomic windows in bins of low and high diversity, or recombination, by the median value. Then, we calculated the number of top 3% and 1% windows with highest introgression proportions in both bins and performed a Pearson's Chi‐squared test with Yates' continuity correction (to correct for potential Type I error when dealing with small expected cell counts) between the top introgression regions and either diversity or recombination.

## 3 Results

### 3.1 Whole‐Genome Data Recovers a Well‐Supported Species Tree

The whole‐genome neighbour‐joining (NJ) tree clusters samples per species and revises the earlier phylogenies (Figure 1; Goropashnaya et al. 2012; Goropashnaya et al. 2004; Romiguier et al. 2018; although see Borowiec et al. (2021)). The species tree is supported by the windowed gene tree inference, where the inferred species tree occurs as the most common topology, representing > 10% or > 16% of all tree topologies for windows with a minimum of 20 or 50 variant sites, respectively (Figure S3). The species tree shows that the *F. rufa* group consists of two sister species pairs— *F. aquilonia* – *F. lugubris* and *F. polyctena* – *F. rufa* —as well as a more distantly related, early‐divergent *F. pratensis* . In the NJ tree, long individual branch lengths relative to internal branches separating the different species indicate high amounts of unsorted genetic variation. Individuals of each species form monophyletic groups with 100% bootstrap support, except for *F. polyctena* that shows nested clustering, which likely suggests geographical differences in the *F. polyctena* introgression history. The neighbour‐joining network (Figure S1) further shows that *F. rufa* and *F. polyctena* cluster by species regardless of their geographic origin, resolving a question left open in earlier work (Satokangas et al. 2023).

### 3.2 Young Species Group With no Signal of Genome‐Wide Barriers With Summary Statistics

**FIGURE 2:**
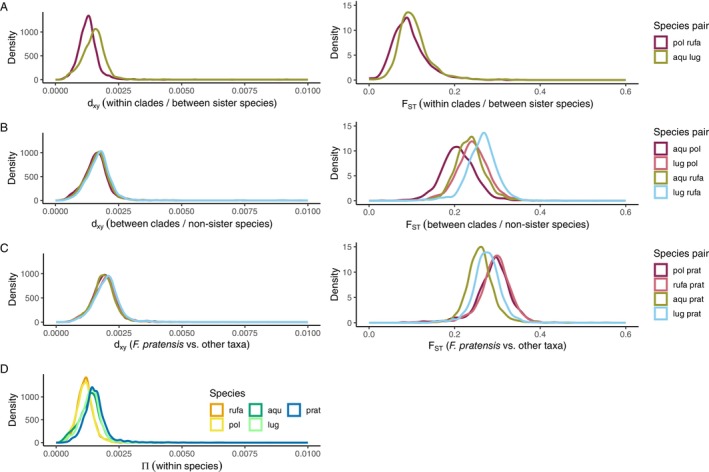
Patterns of genome‐wide variation. Levels of divergence (*d*_xy_), differentiation (*F*_ST_), and diversity (*π*) within the *F. rufa* group computed in 100 kb genomic windows demonstrate an early stage of divergence. *d*_xy_ and *F*_ST_ are compared between (A) sister species, (B) clades (non‐sister species), and (C) the basal *F. pratensis* and all other species. (D) presents within‐species diversity. Five individuals per species are used, shown in NJ‐tree (Figure 1B) with circle symbols.

We found low levels of diversity across the whole group as measured with *π*, the average number of nucleotide differences per site within each species. Slightly lower values were observed in the southerly distributed species *F. rufa* and *F. polyctena* , in comparison to the others (Figure 2D). *d*_xy_, the average number of nucleotide differences per site between species, suggests that the group has started to diverge yet the divergence is still low (*d*_xy_ marginally higher than *π*; Figure 2A–C, left panel). This divergence resembles the amount of within‐species diversity, particularly in the sister species pairs. *F*_ST_, which reflects the allele frequency differences between the taxa, increases for the most part with phylogenetic distance, yet there are differences between species pairs with equally distant phylogenetic histories (Figure 2A–C, right panel).

**FIGURE 3:**
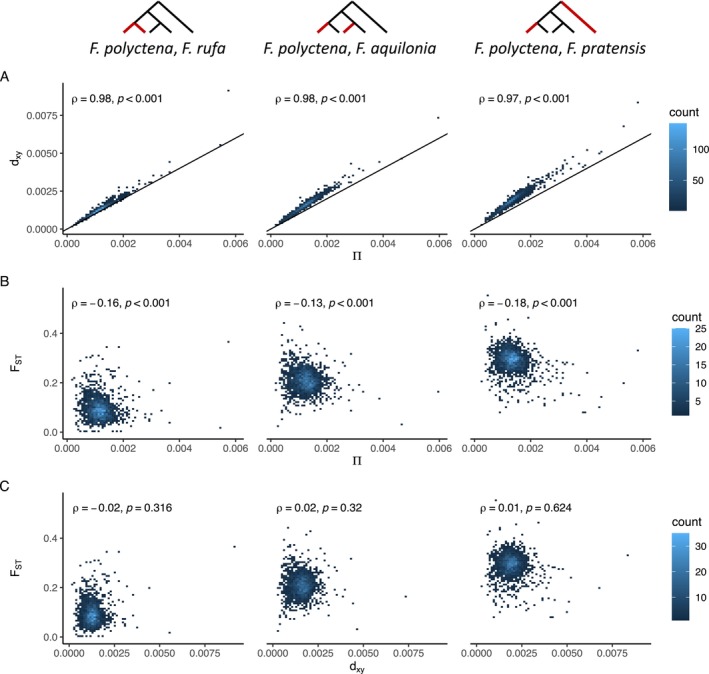
Genome‐wide correlations between population genetic parameters along the early stages of speciation continuum. Correlations between population genetic parameters are shown for three species pairs with increasing phylogenetic distance (for the rest of the comparisons, see Figures S4–S6). The values are computed in 100 kb genomic windows. (A) Divergence (*d*_xy_) is highly correlated with diversity within species (*π*, averaged for the species compared) in all species pairs. *d*_xy_ is higher than average *π* in all species pairs and this difference strengthens with phylogenetic distance. A diagonal line with slope 1 is drawn to help see how *d*_xy_ and *π* relate. (B) Small and similar proportion of differentiation (*F*_ST_) is explained by diversity (*π*), and this pattern does not change despite increasing phylogenetic distance between species pairs. (C) As differentiation (*F*_ST_) between species pairs increases, so does divergence (*d*_xy_), however, there is no indication of positive genome‐wide correlation in the introgressing species pairs (left and middle column). Correlation tests are performed with Spearman's correlation.

Among the five *F. rufa* group species, divergence is highly correlated with within‐species diversity (Spearman's rho = 0.97–0.99, *p* < 0.001 in all comparisons), as expected for young species in which divergence still reflects ancestral diversity. In all comparisons, divergence is, however, always higher than diversity and this difference increases with phylogenetic distance (Figures 3A and S4). Differentiation, on the other hand, is only to a very small extent explained by diversity (Spearman's rho = −0.08 to −0.32, *p* < 0.001 in all comparisons (Figures 3B and S5)) and the strength of this correlation is not related to the phylogenetic distance. Furthermore, when inspecting the species pairs in which we detect significant genome‐wide introgression with the *f*‐branch statistic ( *F. polyctena* and *F. rufa* , and *F. polyctena* and *F. aquilonia* , see below 3.3.), we find no genome‐wide correlation between divergence and differentiation (Figures 3C and S6) that would be expected in the presence of genome‐wide gene flow barriers in species diverging with gene flow.

### 3.3 Introgression at Genome‐Wide and Local Scale

**FIGURE 4:**
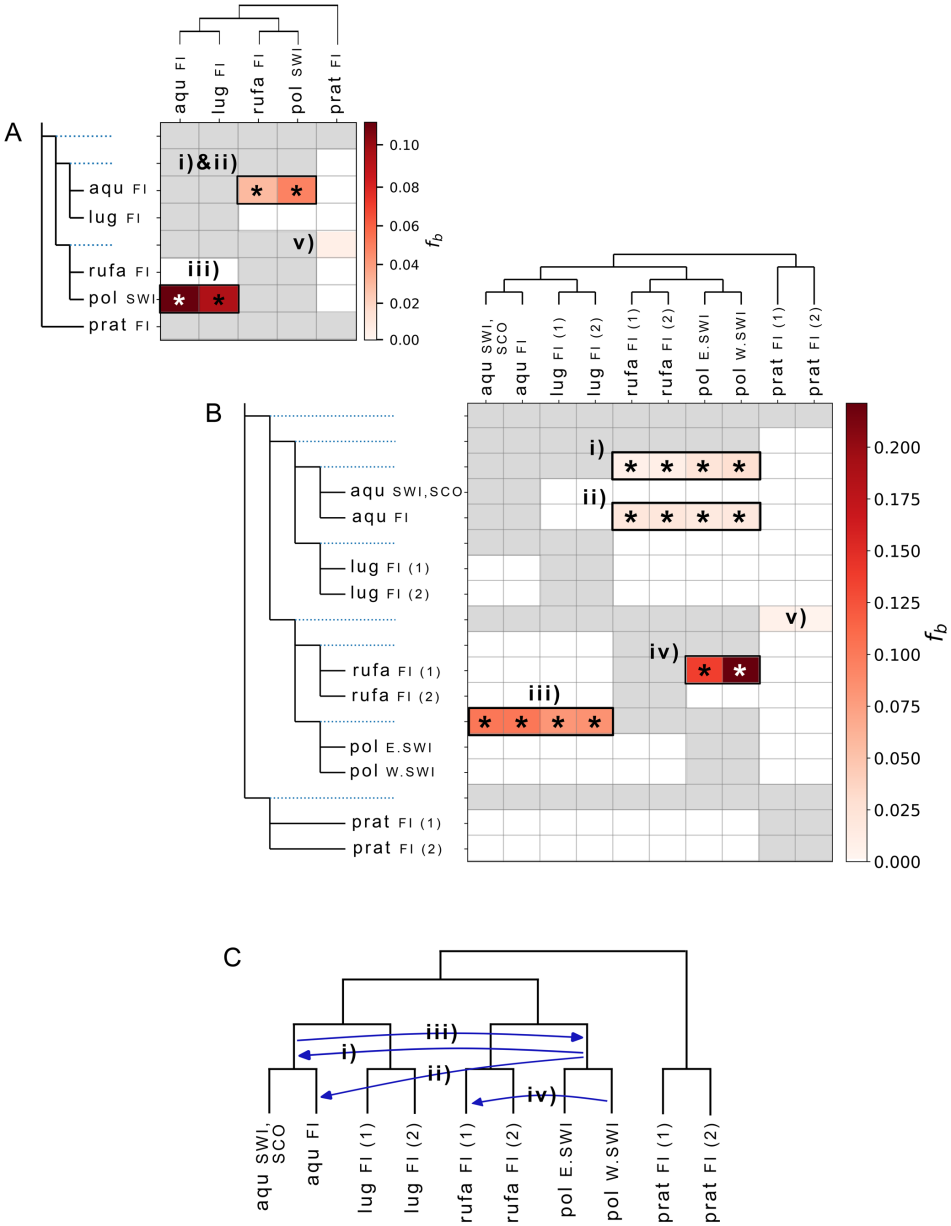
Introgression between species. *f*‐branch statistic demonstrates introgression in at least two species pairs. (A) The simplest scenario, one tree tip per species, suggests bidirectional introgression between *F. polyctena* and *F. aquilonia* (i), (ii), and (iii). Inference of ancestral or within‐clade introgression is not possible in this tree with one tip per species. (B) *f*‐branch statistic computed for a tree, where each species is split according to clustering in the species tree reveals additional introgression events and provides more detailed information. Following the most parsimonious interpretation (i) introgression from *F. polyctena* has occurred into the ancestor of Swiss and Finnish *F. aquilonia* populations, and (ii) it has continued into the Finnish *F. aquilonia* after the geographical split of *F. aquilonia* populations. Additional introgression has occurred from *F. polyctena* into *F. rufa* (iv). (v) Potential signal of introgression between *F. pratensis* and the *F. polyctena/F. rufa* clade is slightly below significance threshold. Grey data points indicate tests that were not possible given the phylogeny. The horizontal patterns may arise from correlated ancestries between sister taxa of the introgression source, which provides the most parsimonious explanation for the introgression events. Introgression between *F. polyctena* and the ancestor of the two *F. rufa* groups cannot be tested but is likely given the extent of their hybridisation Europe‐wide. Asterisks (*) indicate significant test results (z‐score > 3). (C) A graphical summary of the inferred most parsimonious introgression events.

We found evidence for introgression between two species pairs: sister species *F. polyctena* and *F. rufa* , and non‐sister species *F. aquilonia* and *F. polyctena* (Figure 4A–C). In *F. aquilonia* and *F. polyctena* , the introgression was inferred to be bidirectional (Figure 4A,B: i, ii, and iii) consistent with demographic models of their recent history (Portinha et al. 2022). For *F. polyctena* and *F. rufa*, the detected excess of introgression between *F. polyctena* and part of the *F. rufa* populations suggests introgression at least from *F. polyctena* into *F. rufa* (Figure 4B: iv), while bidirectional introgression cannot be ruled out.

More specifically, the results suggest that introgression from *F. polyctena* into *F. aquilonia* has occurred into the ancestor of Swiss and Finnish *F. aquilonia* populations and continued into the Finnish but not Swiss *F. aquilonia* after their geographical split. The z‐score for introgression between *F. pratensis* and the clade of *F. polyctena* and *F. rufa* (2.8 and 2.7 in Figure 4A,B, respectively) remained slightly below the significance threshold (z‐score 3).

**FIGURE 5:**
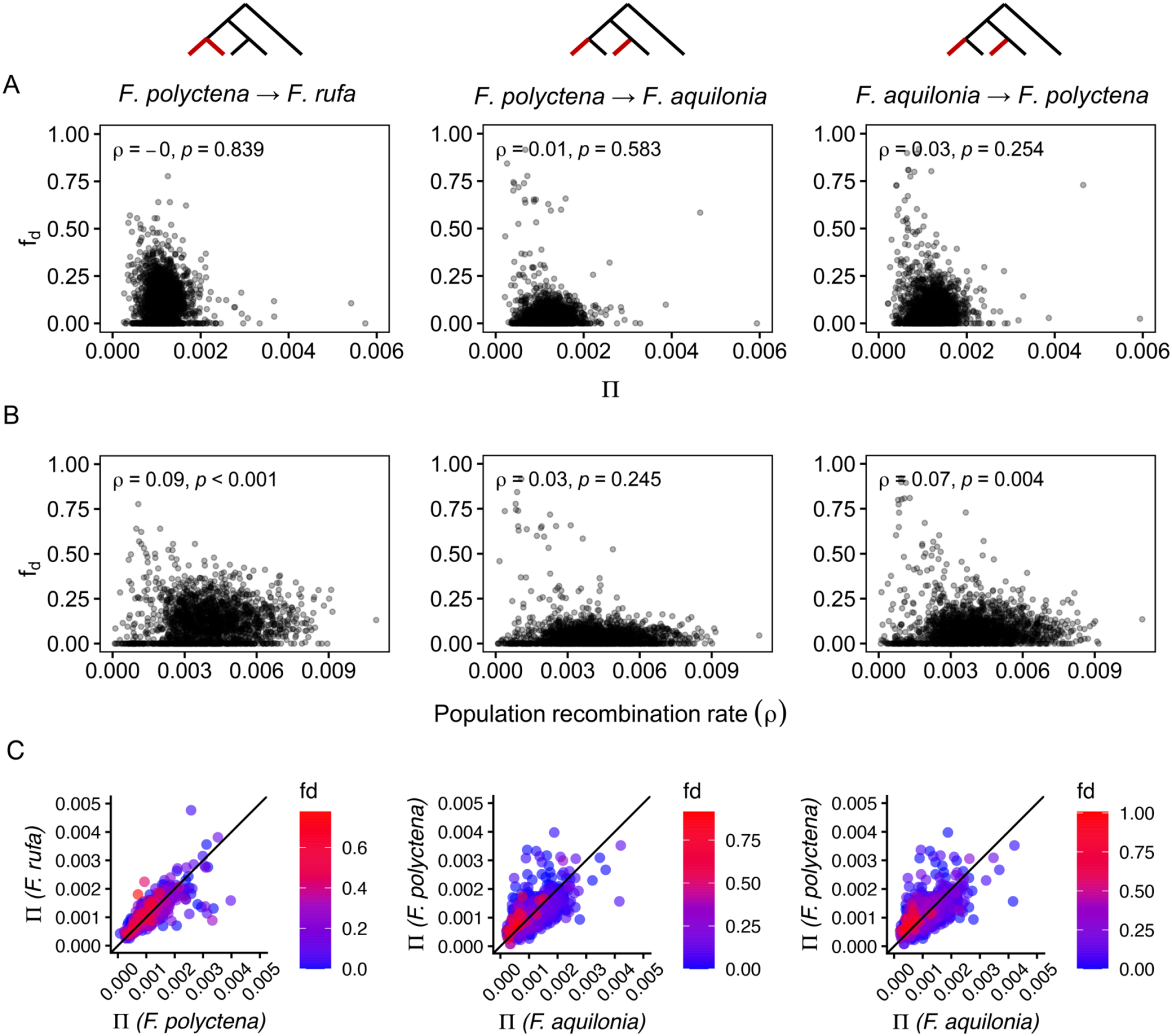
Genome‐wide correlations between introgression (*f*_d_), diversity (*π*), and recombination (*ρ*). In species pairs with detected significant introgression, genome‐wide correlation between introgression and diversity or recombination is non‐existent or very weakly positive. Computed in 100 kb windows. (A) Correlation between diversity (average of the two species) and introgression, (B) Correlation between population recombination rate (average of *F. aquilonia* and *F. polyctena* ) and introgression, (C) Correlation between diversities within each species, coloured with the proportion of introgression. Diagonal line with slope 1 helps to compare the diversity between gene flow source and recipient species in regions with high proportion of introgression in panel C. Correlation tests are performed with Spearman's correlation.

At the whole‐genome level, we detected no significant correlation between the estimated proportion of introgression (*f*_d_) and diversity (*π*) (Figure 5A), and no or a very weak positive correlation between *f*_d_ and recombination (Figure 5B) (results remained similar when performing the analysis with 50 kb and 100 kb windows). This suggests that, in contrast to other systems, such as *Heliconius* butterflies and Swordtail fish (*Xiphophorus*; Martin et al. 2019; Schumer et al. 2018), the overall impact of introgression in these young wood ant species is neutral.

Moreover, locally in the genome, the relation between high proportion of introgression and both diversity (averaged for the species in question) and recombination was significant in both *F. aquilonia* – *F. polyctena* species pair, and *F. polyctena* – *F. rufa* species pair, when both 1% and 3% top introgression outlier windows were examined (Table 2). In all tests, high introgression windows had lower diversity and lower population recombination rate than expected by chance. In addition, to examine the underlying reason behind the association of low diversity and high introgression, we examined visually species‐specific diversity in relation to estimated introgression and inferred that the highest estimated proportion of introgression is often located in regions with relatively low diversity in both the introgression source and recipient species, although variation in this does exist (Figure 5C).

**TABLE 2:** Association of high introgression and diversity or recombination.

Introgressing species and the direction of gene flow	% Of top outlier introgression windows (*fd* threshold)	Count of the outlier introgression windows in each bin		Diversity (*π*)			Population recombination rate (*⍴*)		
		*π*	*⍴*	*X* ^2^	df	*p*	*X* ^2^	df	*p*
		Low/high	Low/high						
pol → aqu	1% (0.438)	16/4	19/1	6.1249	1	0.013*	14.619	1	< 0.001**
	3% (0.165)	49/9	47/11	27.079	1	< 0.001**	21.813	1	< 0.001**
aqu → pol	1% (0.535)	19/1	19/1	14.619	1	< 0.001**	14.619	1	< 0.001**
	3% (0.308)	49/9	48/10	27.079	1	< 0.001**	24.375	1	< 0.001**
pol → rufa	1% (0.466)	15/5	19/1	4.0929	1	0.043*	14.603	1	< 0.001**
	3% (0.358)	41/18	38/21	8.4652	1	0.004**	4.4774	1	0.034*

*Note:* Locally in the genome, high proportions of introgression are associated with low diversity and low recombination (i.e., below median diversity or recombination value). The data is computed in genomic windows of 100 kb and tested with chi‐squared test, N(aqu pol) = 1930, N(pol rufa) = 1909. pol = *F. polyctena* , aqu = *F. aquilonia* , rufa = *F. rufa* .

* Significant at 0.05 level.

** Significant at 0.005 level.

Genomic regions with lower population recombination rates and diversity are likely to experience higher drift (lower effective population size, N_e_) and less independence among loci (lower recombination), which may increase genealogical noise and therefore the number of *f*_d_ outliers (Martin et al. 2015). To test whether the enrichment of introgression outliers in low diversity and low recombination regions can be explained by noise alone, we investigated whether there is also an enrichment of negative *f*_d_ values in these regions. Negative *f*_d_ should be a rare occurrence caused by genealogical noise.

Neither of the introgressing species pairs showed consistent enrichment of negative *f*_d_ outliers in regions of low *π*, but there was an enrichment of negative *f*_d_ outlier windows in regions of low *ρ* (Table S4).

As an additional test, we inspected the levels of *f*_d_ in three species pairs with no detected significant genome‐wide introgression. A visual inspection showed a lesser amount of high introgression outlier windows in regions of low *π* or low *ρ* in comparison to species pairs with significant genome‐wide introgression (Figure S7; panel A for *π*, and B for *ρ*). This supported the result that the high *f*_d_ values in the introgressing pairs cannot be explained by noise alone.

These results together suggest that the enrichment of introgression observed in regions of low diversity cannot be explained by a statistical artefact of elevated drift, whereas at least some of the enrichment in regions of low recombination may be caused by increased genealogical stochasticity. The genome‐wide correlations between *f*_d_ and *π* or *ρ* remained the same despite the inclusion of negative *f*_d_ values, overruling the possibility that high *f*_d_ values caused by noise would have affected our whole‐genome correlations (Figure S8, Table S4).

## 4 Discussion

The role of introgression in adaptive evolution is increasingly acknowledged across taxa with different levels of divergence (Edelman and Mallet 2021). Recent theoretical work revisits the conventional expectation that introgression may only remain in the genome in regions of high recombination, seen as a positive correlation between recombination and introgression. This expectation arises because introgression is assumed to be mostly deleterious and is therefore selected against in low recombining regions, while in high recombining regions, recombination can separate small adaptive or neutral parts from the introgressed material, allowing them to be maintained in the population (Veller et al. 2023). The recent work suggests that in young species large introgressing blocks may remain intact because they may instead have positive or neutral fitness effects (Dagilis and Matute 2023). Using whole‐genome resequencing data from *Formica rufa* group we find at most a weak positive genome‐wide correlation between introgression and recombination despite polygenic barriers described in earlier work (Kulmuni and Pamilo 2014; Kulmuni et al. 2020). This contrasts with findings from more diverged taxa with relatively less introgression in low recombining regions (Martin et al. 2019; Schumer et al. 2018). Hence, our results are in line with the novel theoretical expectations (Dagilis and Matute 2023). Moreover, we identify local genomic signatures of introgression that may have been favoured by selection. When hybridization and introgression have the potential for both benefits and costs, their long‐term impacts for species are challenging to predict. Our findings shed light on the role of introgression in a taxon group, where previous research has inferred both polygenic gene flow barriers and deleterious fitness consequences of hybridisation (Kulmuni and Pamilo 2014; Kulmuni et al. 2020), but also potential adaptive benefits of hybridisation (Martin‐Roy et al. 2021; Satokangas et al. 2023; Nouhaud, Martin, et al. 2022).

### 4.1 No Strong Genome‐Wide Signature of Deleterious Introgression

Our data suggests there is at most a weak positive genome‐wide association between recombination and introgression. This deviates from expectations when species barriers are polygenic and introgression is selected against at many loci dispersed throughout the genome. Instead, it suggests that introgression in the *F. rufa* species group is largely neutral, or that the fitness benefits of large introgressing blocks may outweigh their fitness costs. These fitness benefits may arise from a direct fitness advantage of the introgressed material, or from the preservation of combinations of coadapted alleles in the introgressed haplotypes. Increased introgression in regions of low recombination after secondary contact has been observed in *Drosophila melanogaster* (Duranton and Pool 2022; Pool 2015; Dagilis and Matute 2023) and European crows (Gwee et al. 2025). Similarly, research on primates suggests a lack of correlation between recombination and introgression in recently diverged species pairs (Jensen et al. 2023).

We inferred introgression between two species pairs; the sister species *F. rufa* and *F. polyctena* , and non‐sister species *F. aquilonia* and *F. polyctena* . Hybridisation between both species pairs is prevalent in nature (Seifert 2021; Seifert et al. 2010; Satokangas et al. 2023). There are two plausible reasons that can explain the very weak, or lack of positive genome‐wide correlation between introgression and recombination found here. First, it could be that the polygenic barriers have not evolved yet. Barriers to gene flow that would be present across the genome could result in selection against large introgressing blocks and hence, more effective selection against introgression in regions of low recombination rate. This may be likely in the case of the closely related sister species *F. rufa* and *F. polyctena* . However, it is contrary to findings suggesting polygenic barriers in the non‐sister species *F. aquilonia* and *F. polyctena* (Kulmuni and Pamilo 2014; Kulmuni et al. 2020; Heidbreder et al. 2026), in which recessive incompatible loci scattered across the genome were shown to cause hybrid male mortality (Kulmuni et al. 2010). In the non‐sister species, perhaps a more likely explanation would be that the benefits of introgression outweigh the fitness costs of genetic incompatibilities that are revealed in introgression. Introgression has a beneficial fitness effect if it delivers adaptive alleles (Edelman and Mallet 2021), masks and helps purging deleterious mutations like in brook charr (Leitwein et al. 2019) and humans (Harris and Nielsen 2016), or when coadaptation within introgressing haplotypes is costly to break, as shown by theoretical work (Dagilis and Matute 2023). We consider the alternative that the benefits of introgression outweigh their costs more likely at least in the case of the non‐sister species *F. aquilonia* and *F. polyctena* .

Interestingly, natural selection is unstable at some of the barrier regions between *F. aquilonia* and *F. polyctena*, switching from acting against hybridisation to favouring it across a 10‐year period and pointing towards environment‐dependent incompatibilities (Kulmuni et al. 2020; Martin‐Roy et al. 2021). We do not know yet how such instability affects the long‐term fitness consequences and signatures of introgression. However, this question is recognised in recent research, and for instance in flowering plants, environmental fluctuations are suggested to affect the amount of gene flow and reproductive isolation between recently diverged subspecies (Sianta et al. 2024).

### 4.2 Potential Signatures of Positive Selection in Regions of High Introgression and the Consequences of Multi‐Species Introgression

Recent research has provided evidence that introgression is an important source of genetic variation in adaptive evolution (reviewed by Edelman and Mallet (2021)). We detected low nucleotide diversity in regions of high introgression, suggesting that some introgressed alleles may have been favoured in both the sister and non‐sister species pairs. In these outlier regions, relatively low diversity was found in both the introgression source and recipient species. This suggests that both the transfer of adaptive alleles and purging or masking of genetic load (deleterious recessive alleles) would be biologically reasonable causes. While it needs to be kept in mind that the reduction of diversity by background selection in these regions cannot be here teased apart, earlier work in wood ant hybrids supports both adaptive scenarios. Specifically, populations of hybrids between the non‐sister species *F. aquilonia* and *F. polyctena* show predictable ancestry sorting: consistent with purging of genetic load, the hybrids had enriched ancestry from the higher effective population size species in coding regions. Furthermore, consistent with the transfer of adaptive alleles, the hybrids were likely to fix haplotypes that had signatures of positive selection in either *F. aquilonia* or *F. polyctena* (Nouhaud, Martin, et al. 2022).

Eusocial Hymenoptera typically have low effective population sizes (Weyna and Romiguier 2021), which may lead to the accumulation of genetic load (Kim et al. 2018). Our results show low genome‐wide nucleotide diversity in all studied species, which may be a manifestation of low effective population size in the *F. rufa* group. Even if both the introgression source and recipient species have low effective population sizes that cause the accumulation of deleterious mutations, these mutations may occur in different sites and introgression may be beneficial, as has been suggested as a possible factor shaping the landscape of introgression between Neanderthals and humans (Harris and Nielsen 2016). The long‐term impacts of such a process are, however, difficult to predict. Neanderthal alleles that were initially favoured due to the masking of deleterious recessives in humans could later be selected against due to the greater genetic load carried by Neanderthals (Schumer et al. 2018; Edelman and Mallet 2021).

Introgression may, on the other hand, represent a source of locally adaptive alleles. It has been suggested that the hybrids between the non‐sister species *F. aquilonia* and *F. polyctena* may benefit from combining the climatic adaptations from the divergently adapted parental species. This may help them cope with a changing climate (Martin‐Roy et al. 2021; Satokangas et al. 2023). From the perspective of the hybridising species, the northern cold‐adapted *F. aquilonia* is suggested to suffer from warm winters (Sorvari et al. 2011) and hence could benefit if introgression from *F. polyctena* mediated tolerance towards warm conditions. Introgression with adaptive potential in the context of climate change is topical and has been detected, for instance, in red oaks, in which it is associated with drought tolerance and influenced by habitat conditions (Khodwekar and Gailing 2017).

Other reasons for the local association of low diversity and high introgression other than adaptive introgression need to be considered as well. Signals of introgression may be biased by stochastic fixation in regions of tight linkage and low effective population size. Extensive simulations (Martin et al. 2015) show that the *f*_d_ statistic used here outperforms other comparable *f*‐statistic estimators in reliability when recombination is low. However, we cannot fully rule out that stochastic fixation would explain the enrichment of introgression outliers in regions of low recombination and low diversity. Additionally, low diversity in both the recipient and the source species in a region of high introgression might be caused by long‐term strong background selection instead of adaptive introgression. These alternative explanations will need to be addressed in future work. Our current approach with 100 kb genomic window size does not reveal fine‐scale variation in introgression and diversity along the genome. Hence, to reveal potential drivers of high introgression in regions of low diversity, it will be essential to study these genomic regions in finer detail.

Sometimes introgression may involve more than two taxa. These so‐called interbreeding complexes have been reported in a variety of organisms from plants to invertebrates and vertebrates (see, e.g., Grant and Grant (2020) and references therein), but their evolutionary consequences are poorly understood. Our results show that the wood ants form an interbreeding complex: introgression was detected in two species pairs— *F. rufa* — *F. polyctena* and *F. aquilonia* — *F. polyctena* —such that *F. polyctena* acts as a shared hybridising partner (presented in Figure 4B).

The introgressing alleles may therefore potentially pass through all these three species. Our data suggests that if introgression is transmitted across all three species, it could flow from northerly distributed, cold‐adapted *F. aquilonia* through southerly distributed, warm‐adapted *F. polyctena* , to southerly distributed *F. rufa* . The other direction (from *F. rufa* through *F. polyctena* to *F. aquilonia* ) could also be possible, given bidirectional gene flow between the non‐sister species and earlier inferences of gene flow in the opposite direction in the sister species (Seifert et al. 2010). Hence, gene flow between multiple taxa could allow alleles to be transferred between taxa that do not otherwise hybridise.

We cannot exclude the possibility of direct introgression also between *F. aquilonia* and *F. rufa* , as hybrid populations between them exist (Satokangas et al. 2023). However, our results indicate that if such introgression occurs, it is not as strong as that between the other species pairs. We do not yet know the consequences of this triad hybridisation and introgression. Introgressing species may act as “genetic bridges” that deliver and hence increase genetic variation across taxa, as seen in the Galapagos finches (Grant and Grant 2020), and hybridisation among multiple lineages may facilitate extremely fast adaptive radiations, as seen in African cichlids (Meier et al. 2023).

### 4.3 Differentiation and Divergence Follow Patterns Expected for Young Species

Our results are in line with expectations for young species, as we detect low overall divergence, high positive correlation between divergence and diversity, and weak correlation between differentiation and diversity (Burri 2017). Other closely related taxa, like the *Ficedula* flycatchers, show similar divergence, differentiation, and diversity estimates as well as similarly strong positive correlation between diversity and divergence (Chase et al. 2021). In addition, in the wood ants, the extensive hybridisation detected previously (Satokangas et al. 2023) and the high amount of unsorted genetic variation revealed by our NJ‐tree (long individual branch lengths relative to internal species' branches) are typical for young taxon groups.

Our data indicated that gene flow barriers do not manifest as a positive whole‐genome correlation between divergence (*d*_xy_) and differentiation (*F*_ST_) in this group. When speciation occurs with gene flow, genomic regions that restrict the gene flow are expected to show both elevated differentiation and elevated divergence (Cruickshank and Hahn 2014). In the presence of polygenic gene flow barriers, a positive genome‐wide correlation between divergence and differentiation would be expected (Shang et al. 2023). Hence, such correlation could have been likely especially in the non‐sister species *F. aquilonia* and *F. polyctena* that have diverged with gene flow (Portinha et al. 2022), show hybrid mortality and harbour barrier loci (Kulmuni and Pamilo 2014; Kulmuni et al. 2020) that are distributed genome‐wide (Heidbreder et al. 2026). A likely explanation is that there has been insufficient time for divergence to develop a detectable signal at the whole‐genome level. This explanation is supported by the fact that we found genome‐wide divergence to still resemble ancestral diversity in the whole *F. rufa* group.

Previous work has presented opposing views about the phylogenetic relationships in the *F. rufa* group. The species tree we constructed in this study consists of two sister species pairs and a more basal *F. pratensis* . It is in line with a recently constructed mitochondrial network (Satokangas et al. 2023) and an earlier nuclear phylogeny that contains four out of our five study species (Borowiec et al. 2021). However, it revises other formerly constructed mitochondrial (Goropashnaya et al. 2012, 2004) as well as nuclear (Romiguier et al. 2018) phylogenies. These likely suffer from low sample size (from one to a maximum of a few individuals per species) that may result in incorrect phylogeny if the individual used happens to have hybrid origin; a plausible possibility given the high hybridisation in the *F. rufa* group (Seifert 2021; Satokangas et al. 2023). The monophyly of *F. rufa* , despite sampling from different geographic locations, confirmed that it forms a distinct gene pool from its sister species *F. polyctena*, which remained as an open question in previous work (Satokangas et al. 2023).

## 5 Conclusions

Using whole‐genome sequencing data, we have inspected divergence and introgression in extensively hybridising wood ants. We showed that there is no strong genome‐wide signature of deleterious introgression—a finding that concerns both the sister and non‐sister species in this young species group. This was revealed by at most a weak correlation between recombination and introgression, which is in line with recent theoretical work. We also detected local signatures that may be caused by positive selection acting for the introgressed material. Previous work in wood ant hybrids gives reason to suggest that introgression could have the potential to help the *F. rufa* group species cope with genetic load or facilitate their survival in a changing climate. Further work on verifying and investigating the signature of adaptive introgression in more detail will shed light on the evolutionary impacts of introgression in the wood ants. This work highlights the notion that evolution towards full reproductive isolation may not promote species persistence in the long run. Instead, divergent taxa may gain by not completing speciation (Barton 2020), although the long‐term impacts of introgression are complex to predict.

## Author Contributions

Original idea: I.S., J.K.; Conceptualisation: I.S., H.H., J.K.; Methodology and Investigation: I.S., S.H.M., B.S., T.P., R.S.; Visualisation: I.S.; Writing and editing: I.S., S.H.M., B.S., T.P., R.S., H.H., J.K.

## Funding

The research of BS and RS was co‐financed by tax money on the basis of the state budget passed by the Sächsischer Landtag according to the Antragsnummer 100590787 of the Sächsische Aufbaubank issued 3 August. JK was funded by the Academy of Finland grant 328961 and 346805. IS was funded by the Doctoral Programme in Integrative Life Science, University of Helsinki, and a grant from the Alfred Kordelin Foundation.

## Ethics Statement

This research complies with all applicable laws on sampling from natural populations.

## Conflicts of Interest

The authors declare no conflicts of interest.

## Supporting information


**Figure S1:** Neighbour‐joining network performed with 9.816 variants, following pipeline in Satokangas et al. (2023) to demonstrate how new sequenced samples relate with previously published ones.
**Figure S2:** An illustration of the trees used for studying introgression with the f‐branch statistic.
**Figure S3:** Most commonly inferred tree topologies with TWISST, computed from coding sequences with minimum A) 50 variant sites per each genomic window (200 windows), and B) 20 variant sites per window (1475 windows).
**Figure S4:** Correlations between diversity (π) and divergence (dxy).
**Figure S5:** Correlations between diversity (π) and differentiation (FST).
**Figure S6:** Correlations between divergence (dxy) and differentiation (FST).
**Figure S7:** Correlations between introgression (fd), and diversity (π) or recombination (ρ) with negative fd values included.
**Figure S8:** Correlations between introgression (fd), and diversity (π) or recombination (ρ).

**Table S1:** Detailed information on sampling.
**Table S2:** Mapping rates for all individuals used in this study.
**Table S3:** Per‐individual coverage. Average read depth for the newly sequenced individuals computed from bam files after overlap clipping.
**Table S4:** Association of low negative fd values, and diversity (π) or recombination (ρ).

## Data Availability

Raw sequencing reads and aligned reads (BAM files) are available at the European Nucleotide Archive (ENA) under project accession PRJEB112102. Part of this data has been used in previous studies (https://doi.org/10.1111/mec.16992 and https://doi.org/10.1093/molbev/msaf320). Derived genomic data, including VCF files, analysis input files, and summary outputs, are available on Dryad (DOI: 10.5061/dryad.95x69p91c). Bioinformatic scripts are available on Zenodo (DOI: https://doi.org/10.5281/zenodo.20025904).
