# Supplementary material for: Introgression and Divergence in a Young Species Group
Source: Mol Ecol. 2026 Jun 26;35(12):e70448. doi: 10.1111/mec.70448 (PMC13308388; doi:10.1111/mec.70448)
Supplement: Supplementary file 1 — Figure S1: Neighbour‐joining network performed with 9.816 variants, following pipeline in Satokangas et al. (2023) to demonstrate how new sequenced samples relate with previously published ones. Figure S2: An illustration of the trees used for studying introgression with the f‐branch statistic. Figure S3: Most commonly inferred tree topologies with TWISST, computed from coding sequences with minimum A) 50 variant sites per each genomic window (200 windows), and B) 20 variant sites per window (1475 windows). Figure S4: Correlations between diversity (π) and divergence (dxy). Figure S5: Correlations between diversity (π) and differentiation (FST). Figure S6: Correlations between divergence (dxy) and differentiation (FST). Figure S7: Correlations between introgression (fd), and diversity (π) or recombination (ρ) with negative fd values included. Figure S8: Correlations between introgression (fd), and diversity (π) or recombination (ρ). [file MEC-35-e70448-s001.docx]

## Supplemental Figures (S1-S8)

Satokangas, I., Martin, S. H., Seifert, B., Puukko, T., Schultz, R., Helanterä, H., & Kulmuni, J. Introgression and divergence in a young species group.


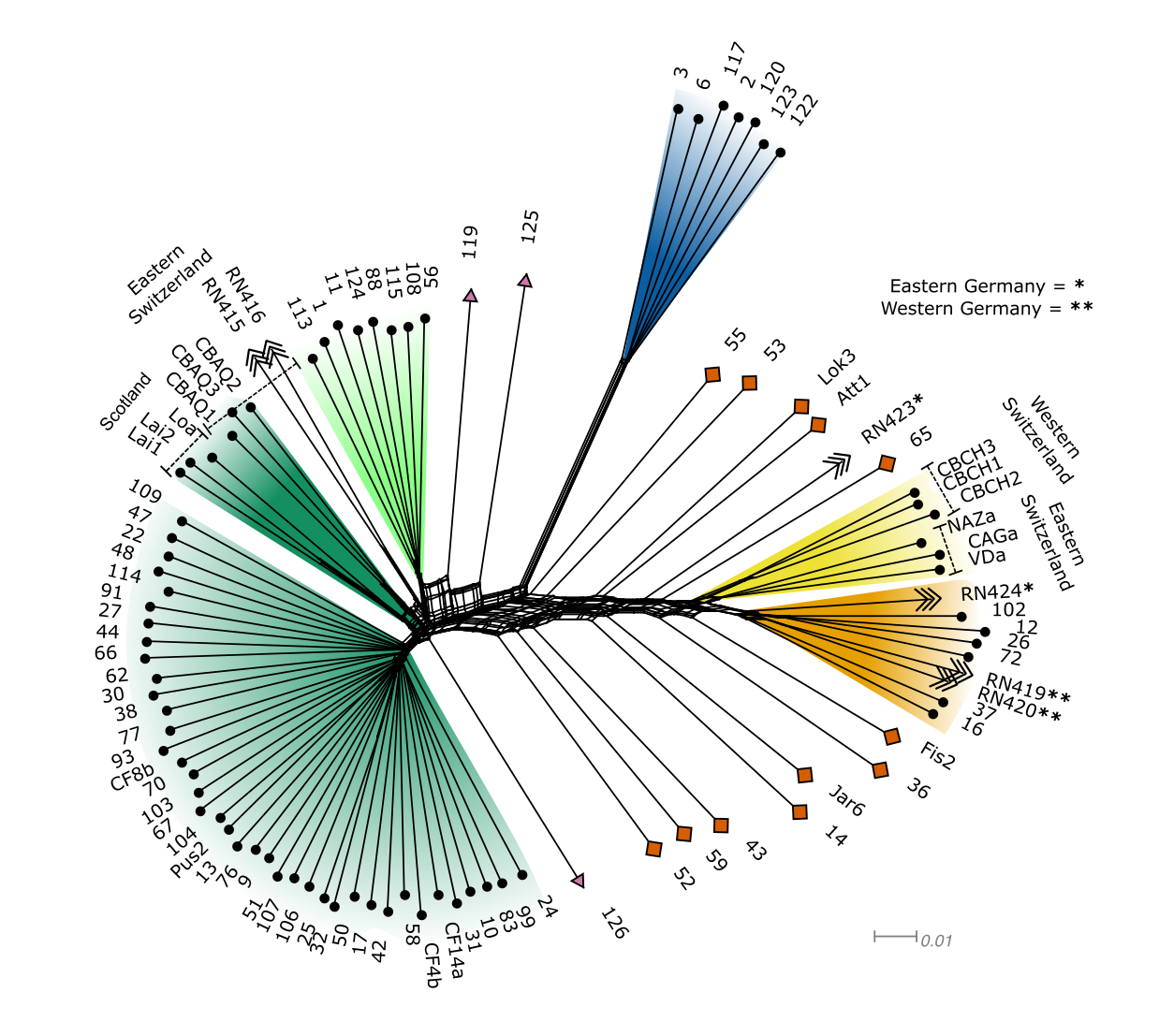


**S1 Fig.** **Neighbour-joining network** performed with 9.816 variants, following pipeline in [(Satokangas et al. 2023)](https://paperpile.com/c/0UKkOa/AcVol) to demonstrate how new sequenced samples relate with previously published ones. Arrowheads indicate newly sequenced individuals. The network especially shows how *F. rufa* and *F. polyctena* cluster by species regardless of geographic origin. All individuals for which geographical location is not given originate from Finland. Newly sequenced individuals RN415, RN416, and RN423 are potentially admixed given their placement in this network. Colours indicate species assignments based on combined morphological and genetic data from this study and previous work [(Satokangas et al. 2023)](https://paperpile.com/c/0UKkOa/AcVol). Dark green = *F. aquilonia*, light green = *F. lugubris*, dark blue = *F. pratensis*, yellow = *F. polyctena*, orange = *F. rufa*, red diamond = admixed individual between *F. aquilonia* and the *F. polyctena/F. rufa* clade, pink triangle = admixed *F. lugubris* individual.

A B

**S2 Fig.** **An illustration of the trees used for studying introgression** with the *f*-branch statistic. **A** A simple tree with one tree tip per species (5 individuals per species and the outgroup). **B** A tree where each species is split into two groups based on clustering in the species tree (2-3 individuals per species and the outgroup). Species are indicated as aqu = *F. aquiloni*a, lug = *F. lugubris*, rufa = *F. rufa*, pol = *F. polyctena*, prat = *F. pratensis*, exs = *F. exsecta* (used as the outgroup). Origin of sampling is indicated as FI = Finland, SWI = Switzerland, SCO = Scotland, E.SWI = Eastern Switzerland, W.SWI = Western Switzerland.

A**
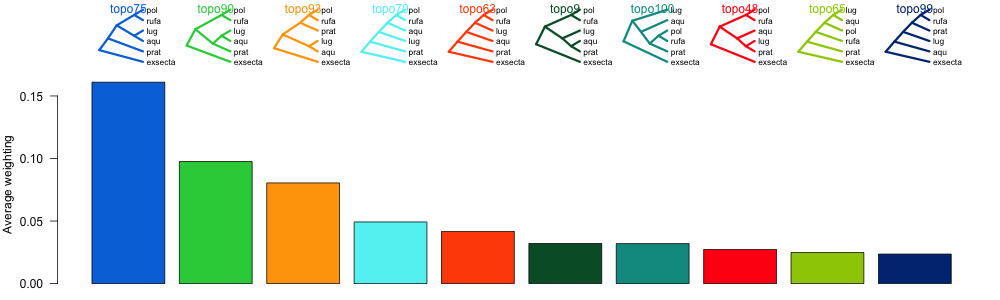
**

B**
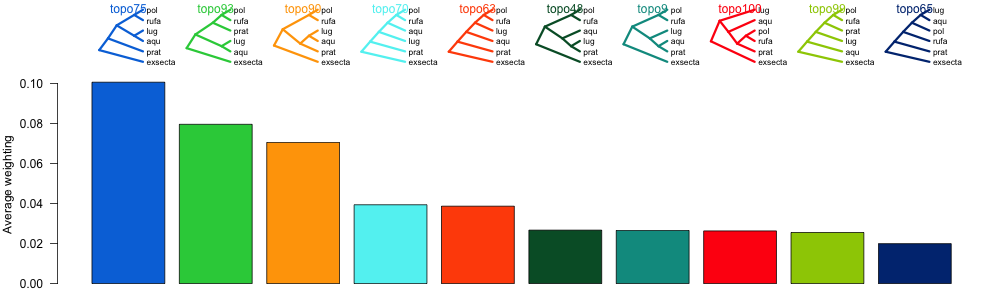
**

**S3 Fig.** **Most commonly inferred tree topologies with TWISST,** computed from coding sequences with minimum **A** 50 variant sites per each genomic window (200 windows), and **B** 20 variant sites per window (1475 windows). Each window is one gene. *F. exsecta* is used as the outgroup. The most frequent topology (topo 75) is the species tree inferred also with the Neighbour-joining tree (Fig 1). The second and third common tree topologies (90, 92) represent likely incomplete lineage sorting as they mirror each other, differing only in clustering *F. pratensis* with either of the sister species clades. Aqu = *F. aquilonia*, lug = *F. lugubris*, prat = *F. pratensis*, pol = *F. polyctena*, rufa = *F. rufa*, exsecta = *F. exsecta*.

**S4 Fig. Correlations between diversity (π) and divergence (d_xy_).** Strong positive correlation between diversity (x-axis) and divergence (y-axis) in all species pairs, computed in 100 kb genomic windows. Diagonal line with slope 1 demonstrates how divergence is higher than diversity, and the difference increases with phylogenetic distance. **A** Sister species pairs, **B** Between-clade species pairs, **C** All species compared to *F. pratensis.* Species are indicated as aqu = *F. aquiloni*a, lug = *F. lugubris*, rufa = *F. rufa*, pol = *F. polyctena*, prat = *F. pratensis.* Correlation tests are performed with Spearman’s correlation.

**S5 Fig. Correlations between diversity (π) and differentiation (F_ST_).** Weak negative correlation between diversity (x-axis) and differentiation (y-axis) in all species pairs, computed in 100 kb genomic windows. **A** Sister species pairs, **B** Between-clade species pairs, **C** All species compared to *F. pratensis.* Species are indicated as aqu = *F. aquiloni*a, lug = *F. lugubris*, rufa = *F. rufa*, pol = *F. polyctena*, prat = *F. pratensis.* Correlation tests are performed with Spearman’s correlation.

**S6 Fig. Correlations between divergence (d_xy_) and differentiation (*F*_ST_).** Stochastic correlation between divergence (x-axis) and differentiation (y-axis) across the *F. rufa* group, computed in 100 kb genomic windows. **A** Sister species pairs, **B** Between-clade species pairs, **C** All species compared to *F. pratensis.* Species are indicated as aqu = *F. aquiloni*a, lug = *F. lugubris*, rufa = *F. rufa*, pol = *F. polyctena*, prat = *F. pratensis.* Correlation tests are performed with Spearman’s correlation.

**S7 Fig. Correlations between introgression (*f*_d_), and diversity (π) or recombination (ρ) with negative *f*_d_ values included.** The higher number of not only positive but also negative *f*_d_ outliers (a ‘funnel-shaped’ data distribution) in some comparisons may be indicative of high *f*_d_ values caused by increased noise. This noise would arise due to higher drift in regions of low effective population size (N_e_), as both π and ρ are affected by N_e_. Species pairs with significant detected introgression are presented. Values are computed in 100 kb genomic windows. **A** Correlation between diversity (average of the two species) and *f*_d_, **B** Correlation between population recombination rate (average of *F. aquilonia* and *F. polyctena*) and *f*_d_. Species are indicated as aqu = *F. aquiloni*a, rufa = *F. rufa*, pol = *F. polyctena.* Correlation tests are performed with Spearman’s correlation.

**S8 Fig. Correlations between introgression (f_d_), and diversity (π) or recombination (ρ).** The high proportions of introgression seen locally in pairs with significant detected introgression (*F. polyctena* & *F. rufa*; *F. aquilonia* & *F. polyctena*, bidirectional) are not seen to a similar extent in control pairs (*F. pratensis* & *F. polyctena*; *F. pratensis* & *F. aquilonia*; *F. lugubris* & *F. rufa*). This indicates that the high *f*_d_ values are not fully explained by noise due to low information in regions of low effective population size. Values are computed in 100 kb genomic windows. **A** Correlation between diversity (average of the two species) and introgression, **B** Correlation between population recombination rate (average of *F. aquilonia* and *F. polyctena*) and introgression. Species are indicated as aqu = *F. aquiloni*a, lug = *F. lugubris*, rufa = *F. rufa*, pol = *F. polyctena*, prat = *F. pratensis.* Correlation tests are performed with Spearman’s correlation.
